# Factors associated with the designation of a health care proxy and writing advance directives for patients suffering from haematological malignancies

**DOI:** 10.1186/1472-684X-13-57

**Published:** 2014-12-11

**Authors:** Sophie Trarieux-Signol, Stéphane Moreau, Marie-Pierre Gourin, Amélie Penot, Geoffroy Edoux de Lafont, Pierre-Marie Preux, Dominique Bordessoule

**Affiliations:** Centre Hospitalier Universitaire, Service d’hématologie Clinique et de thérapie cellulaire, 2 avenue Martin Luther King, 87042 Limoges, France; Centre Hospitalier Universitaire, Centre d’Epidémiologie de Biostatistique et de Méthodologie de la Recherche, 2 avenue Martin Luther King, 87042 Limoges, France

**Keywords:** Haematology, Advance directives, Proxy, Ethics, Health communication, Spirituality

## Abstract

**Background:**

During the last few decades, patients’ rights have been reinforced in many countries by acts of law. Measures now include health care proxies to uphold the doctor-patient relationship and advance directives for end-of-life patients. These could be relevant tools as early as the initial diagnosis of haematological malignancies because of the uncertain disease course. The aim of this research was to assess the factors associated with the designation of a proxy and writing advance directives by patients in a haematology department in France.

**Methods:**

After a specific programme to encourage discussions about end-of-life preferences, we conducted a mixed-methods study comprising retrospective analysis of a random sample of 200 patients’ medical records, crossed with a qualitative analysis of the content of advance directives. Statistical analysis was performed by the RKward V 0.6.1 software with 0.05 denoting significance. The study was performed and presented in accordance with the STROBE guidelines. A thematic analysis of the advance directives was performed by two researchers.

**Results:**

A total of 197 medical records were evaluable. The mean age of the patients was 66 years (range: 18–91). Nearly 2/3 of them (64.5%) designated a proxy, 6.1% wrote advance directives, and 8.1% and 4.6% expressed a wish to meet a religious representative or a volunteer, respectively. The 2-year survival rate was 78.4% [95%CI: 68.2-90.2]. Patients who wrote advance directives were statistically older (p <0.00025). Patients who wrote an advance directive were more likely to have expressed a wish to meet a religious representative (p <0.001) or a volunteer (p = 0.003). Marital status was a significant factor in appointing a proxy (p = 0.04).

**Conclusions:**

To the best of our knowledge, this is the first paper to identify influencing factors for proxies and advance directives in a homogenous population of patients with haematological malignancies. Most patients chose a proxy. However, despite several training programmes for the carers and a care planning programme, few patients wrote advance directives. Our findings suggest that influencing factors are advanced age and a wish to see a religious representative. This study highlights the importance of oral communication about end-of-life issues between carers, patients and their relatives.

**Electronic supplementary material:**

The online version of this article (doi:10.1186/1472-684X-13-57) contains supplementary material, which is available to authorized users.

## Background

Over the last few decades, patients’ rights have been enforced in many parts of the world by acts of law [1–4]. Nevertheless, their implementation within hospital units varies [5–7] according to the pathology and culture, particularly when comparing North America and Europe [8–10], and various publications [11–13] report insufficient uptake both by carers and the general public. Previous studies have focused more on the carers’ theoretical perception of the law [14, 15] rather than the patients’ perception [16]. In France, the "Patients’ Rights and End-of-Life Care" Act dated April 22, 2005 [4], emphasizes the role of the health care proxy (HCP) and advance directives (ADs). However, in 2011, the French National Observatory of the End of Life, described them as "*misunderstood, difficult to use tools"*[13].

To the best of our knowledge, no such studies have been conducted in the context of haematological malignancies (HM). These diseases have a particularly poor prognosis and the risk of dying remains in the foreground throughout the clinical pathway. Moreover, sceptic shock or sudden bleeding can occur during induction therapy or in advanced stage disease. It is thus logical to introduce the concepts of HCP and AD to a patient in this setting.

The main goal of this study was to assess the factors associated with the designation of an HCP and writing ADs by patients presenting with an HM both quantitatively (how many patients) and qualitatively (content of ADs). The secondary objective was to assess the link between these tools and the patient’s wish to meet a volunteer or a religious representative.

## Methods

Additional file 1: Table S1 summarizes the multi-step procedure initiated in 2008 in our centre to implement the 2005 "Patient’s Rights and End-of-Life Care" Act. Our programme aims to inform patients and carers about HCPs, ADs and to encourage discussions about end-of-life preferences. Carers were trained to present the concept of an HCP and AD to the patient and we revised the patient welcoming procedure. All the patients are asked to complete the "*patient information form"* on arrival. They have the choice to designate an HCP, write an AD and express a wish to meet a volunteer or a religious representative. The AD that we use comprises very few instructions so as not to influence the patient’s choices and encourage the patient to express him/herself freely. We used a mixed-methods approach to collect the data and combine quantitative and qualitative methods to integrate the various relevant perspectives to study HCP and ADs.

### Sample selection

Two hundred medical records of patients presenting with an HM and treated in the Haematological Unit of a University Hospital (Limoges, France) from June 1, 2008 to April 30, 2012 were randomly drawn. Data was extracted by a clinical research engineer using an abstraction protocol to perform a retrospective descriptive quantitative analysis [17]. Demographic and study data were collected from the patient information form which is completed by all patients on their first visit to the centre and kept in their medical records.

Any medical records without this patient information form filled in by the patient or his legal representative were not retained for analysis. Study data included: whether an HCP had been designated, whether an AD had been written and was available, mention of a wish to meet a religious representative or a volunteer. The survival data of the patients was updated on July 5, 2013.

### Statistical analysis

The statistical studies were performed by using the software RKward V 0.6.1 with a significance level of 0.05. Analysis was performed in accordance with the STROBE guidelines. The quantitative variables were studied for their average compared to the standard deviation, or median, and for their interquartile range. The qualitative variables were studied by headcount and percentage.

Binomial univariate logistic regression: the significantly associated variables presenting a predictive factor for HCP or ADs were identified using a multivariate logistic regression model with two dependent variables (HCP or ADs) and independent variables (age, diagnosis, gender, date of the patient information form, marital status, pathology, religious representative and volunteer visit). Relevant variables and those presenting a level of significance below 0.20 in univariate analysis were introduced in the multivariate model. A stepdown procedure was performed with a level of significance below 0.05. For statistical analysis, the variables mentioned above were categorized as follows: age group on diagnosis (under 69 or over 70 years); gender (M/F); date (year) of the patient information form (2008–2009, 2010, 2011–2012); marital status (married or with a partner, single, unspecified); pathology (myeloid pathologies and others, lymphoid pathologies); a wish to meet a religious representative (yes, no, n/a); wish to meet a volunteer (yes, no, n/a).

### Qualitative analysis

#### Data collection

Qualitative data was collected from written ADs available in the patient information form.

#### Data analysis

The aim of the qualitative analysis was not to be exhaustive but rather to provide an overall understanding. All the data were listed on a spreadsheet. No specific qualitative data analysis software was used. The data were analysed according to qualitative content analysis as introduced by Paillet et Mucchielli [18]. More specifically, we performed a thematic analysis of the content of the ADs after identifying the main messages and keywords and assessing recurrence.

The analytical process was conducted by a multidisciplinary research team consisting of a senior haematologist and a clinical research engineer trained in qualitative research, medical law and clinical ethics. Initially, both researchers read the six ADs independently to familiarize themselves with the data. Then they independently analysed each AD and coded data according to the rules. Codes were subsequently compared, contrasted, and grounded in data before being abstracted to related categories. During the analysis there was an ongoing discussion of emerging themes and keywords, and variance of interpretation in the study was resolved through communicative validation.

### Terminology

For this study, HCP was defined in accordance with French regulations [4] as a person chosen by the patient for two types of missions: first, to support him/her in making decisions throughout the healthcare pathway and second, to speak on his/her behalf for future healthcare decisions in case the patient is unable to express wishes him/herself. The HCP can never make a decision in place of the patient.

ADs are a written document by which the patient expresses his/her preferences in case he/she is unable to express them. The patient indicates his/her wishes regarding the end of life and the conditions for limiting or stopping treatment.

Both an HCP and AD are strictly informative and the final decision belongs to the physician.

### Ethics

The Ethics Committee of the University Hospital of Limoges approved the study. According to French health regulations no written informed consent is required as the research is a retrospective observational study on registered data. Oral consent was obtained prior to the research by all participants.

## Results

### Demographic data

A total of 2180 medical records were listed during the study period. Among them, 200 were randomly selected and 197 (98.5%) patient information forms retained for analysis. Three (1.5%) were excluded for missing data. The patients characteristics are summarized in Table 1. There were no significant differences in the number of patients designating an HCP or writing an AD throughout the period (2008–2009, 2010, 2011–2012) from when the information programme was introduced to the end of the study. The sex ratio was 1.26, the average age was 64.4 ± 15.8 [range: 18–91] years and 73 (37.0%) were 70 years or older. A total of 136 (69.0%) and 61 (31.0%) patients presented with malignant lymphoid and malignant myeloid pathologies and others, respectively. The 2-year survival rate of the entire cohort was 78.4% [95% CI: 68.2-90.2]. Sixteen patients (8.1%) expressed a wish to meet a religious representative and nine (4.6%) a volunteer.

**Table 1:** **Characteristics of the cohort of patients**

	Total sample		Health care proxy						Advance directives					
			Yes		No		N/A		Yes		No		N/A	
	n.	%	n.	%	n.	%	n.	%	n.	%	n.	%	n.	%
**Year medical records were studied**														
2008-2009	60	30.5	35	58.3	14	23.3	11	18.3	3	5.0	34	56.7	23	38.3
2010	54	27.4	38	70.4	10	18.5	6	11.1	4	7.4	34	63.0	16	29.6
2011-2012	83	42.1	54	65.1	14	16.9	15	18.1	5	6.0	52	62.7	26	31.3
**Population**	197	100.0	127	64.5	38	19.3	32	16.2	12	6.1	120	60.9	65	33.0
**Gender**														
Male	110	55.8	71	64.5	21	19.1	18	16.4	6	5.5	72	65.5	32	29.1
Female	87	44.2	56	64.4	17	19.5	14	16.1	6	6.9	48	55.2	33	37.9
Sex ratio	1.26		1.27						1					
**Age groups on diagnosis**														
<70	124	63.0	79	63.7	27	21.8	18	14.5	5	4.0	83	66.9	36	29.0
≥70	73	37.0	48	65.8	11	15.1	14	19.2	7	9.6	37	50.7	29	39.7
Median age (years)	66		67						73					
Average age (years) SD (min-max)	64.4 ± 15.8 [18;91]		64.8 ± 14.2 [23;91]						72.5 ± 5.6 [62;83]					
**Marital status**														
Married or with a partner	138	70.1	98	71.0	24	17.4	16	11.6	9	6.5	87	63.0	42	30.4
Single/Widowed	44	22.3	19	43.2	11	25.0	14	31.8	3	6.8	22	50.0	19	43.2
Not filled in	15	7.6	10	66.7	3	20.0	2	13.3	-	-	11	73.3	4	26.7
**Pathologies**														
Lymphoid pathologies^1^	136	69.0	91	66.9	26	19.1	19	14.0	4	2.9	81	59.6	51	37.5
Myeloid pathologies and others^2^	61	31.0	36	59.0	12	19.7	13	21.3	8	13.1	39	63.9	14	23.0
**Wish to meet a religious representative**														
	16	8.1	12	75.0	1	6.3	3	18.8	6	37.5	9	56.3	1	6.3
**Wish to meet a volunteer**														
	9	4.6	6	66.7	1	11.1	2	22.2	3	33.3	5	55.6	1	11.1

^1^Lymphoid pathologies: NHL, Hodgkin’s disease, Hairy cell leukaemia, myeloma, amyloidosis, Waldenstrom’s disease, CLL).

^2^Myeloid pathologies and others: acute leukaemia and myelodysplasia (RAEB), CMML, myeloproliferative disorders (CML, primary myelofibrosis, essential thrombocythemia, polycythemia vera, rare non malignant haemopathies (aplastic anemia, severe autoimmune cytopenias, thrombotic microangiopathies, immune deficiencies.

### Description of the sample of patients who designated a health care proxy

#### Quantitative content analysis

Among the 197 evaluable medical records, 127 patients had designated a HCP (64.5%). The characteristics of these patients are summarized in Table 2. The sex ratio was 1.27 and the average age 64.8 ± 14.2 [range: 23–91] years. Among them, only 12 patients (75.0%) expressed a wish to meet a religious representative and six (66.7%) a volunteer.

**Table 2:** **Designation and identity of the health care proxy**

Designation of a health care proxy	n.	%
Yes	127	**64.5**
No	38	**19.3**
Not filled in	32	**16.2**
*Total*	*n = 197*	***100%***
**Identity of the chosen person**		
Spouse/Partner	76	**60**
Child	30	**24**
Close relative (cousin, nephew, in-law)	5	**4**
Other (doctor, ex-partner)	5	**4**
Sibling	4	**3**
Parent/Grandparent	3	**2**
Unspecified	4	**3**
*Total*	*n = 127*	***100%***

A total of 118 (93.0%) patients chose a relative as their HCP: either the spouse for 76 patients (60.0%) or a descendant for 30 (24.0%). Patients living with a partner, either married or not, preferentially chose their partner (n = 66, 77.5%). The HCP was somebody from outside of the family for four patients (2.6%) and only three (2.0%) chose their physician. Among the seven patients who designated several HCPs, five were married or with a partner (four of them designated their wife or husband as first choice and chose simultaneously their children or stepchildren, and one chose simultaneously two children and one stepdaughter). The two remaining single patients designated three children or two relatives respectively.

The only factor that emerged in univariate analysis as associated with the designation of an HCP was marital status: OR 2.4 [95% CI: 1.0-5.9], p = 0.040. (Table 3).

**Table 3:** **Analysis by univariate binomial logistic regression of the factors associated with the designation of a health care proxy**

Variables		Odds Ratio	95% CI	***p-value***
**Gender**				
	Male	1.0		
	Female	0.9	[0.4-2.0]	*0.944*
**Age**				
	<70 years	1.0		
	≥70 years	1.4	[0.6-3.3]	*0.320*
**Marital status**				
	Single	1.0		
	Married or with a partner	2.4	[1.0-5.9]	*0.040*
**Pathology**				
	Myeloid and others	1.0		
	Lymphoid	0.8	[0.3-1.9]	*0.701*
**Time period**				
	2008-2009	1.0		*0.333*
	2010	1.5	[0.6-3.9]	
	2011-2012	1.5	[0.6-3.6]	
**Wish to meet a religious representative**				
	No	1.0		
	Yes	4.0	[0.7-76.1]	*0.185*
**Wish to meet a volunteer**				
	No	1.0		
	Yes	2.0	[0.3-39.0]	*0.522*

Table 4 summarizes the results of the multivariate analysis. Marital status remained a significant factor: OR 2.4 [95% CI: 1.0-5.9], p = 0.040.

**Table 4:** **Multivariate analysis**

**Result of analysis by multivariate binomial logistic regression using a step-down method of factors associated with the designation of a health care proxy**			
Variables	Odds Ratio	95% CI	*p-value*
**Marital status**			
Single	1.0		
Married or with a partner	2.4	[1.0-5.9]	*0.040*
**Result of analysis by multivariate binomial logistic regression using a step-down method of factors associated with the writing of advance directives**			
Variables	Odds Ratio	95% CI	*p-value*
**Wish to meet a volunteer**			
No	1.0		
Yes	13.0	[2.2-72.1]	*0.003*

### Description of the sample of patients who wrote advance directives

#### Quantitative content analysis

ADs were sampled in the medical records of 12 (6.1%) patients (Table 1). Patients who wrote ADs were equally men or women, with an average age of 72.5 ± 5.6 [range: 62–83] years at the time of diagnosis. Seven (9.6%) were 70 or older and overall this group was statistically older than the rest of the cohort (p = 0.00025). Six of them (37.5%) expressed a wish to meet a religious representative and three (33.3%) a volunteer.

Gender, age and marital status had no predictive value for the writing of ADs, but there were more patients with lymphoid pathologies than with myeloid pathologies (p = 0.027) OR 4.1 [95% CI: 1.2-16.3]. The most significant factors for writing an AD were the wish to meet a religious representative (p < 0.001) or a volunteer (p = 0.003) (Table 5).

**Table 5:** **Analysis by univariate binomial logistic regression of the factors associated with the writing of advance directives**

Variables		Odds ratio	95%CI	***p-value***
**Gender**				
	Male	1.0		
	Female	1.5	[0.4-5.0]	*0.504*
**Age**				
	<70	1.0		
	≥70	3.1	[0.9-11.2]	*0.064*
**Marital status**				
	Single	1.0		
	Married or with a partner	1.1	[0.2-7.8]	*0.874*
**Pathology**				
	Myeloid and others	1.0		
	Lymphoid	4.1	[1.2-16.3]	*0.027*
**Time period**				
	2008 -2009	1.0		*0.947*
	2010	1.3	[0.2-7.2]	
	2011-2012	1.0	[0.2-5.5]	
**Wish to meet a religious representative**				
	No	1.0		
	Yes	14.2	[3.6-59.2]	*<0.001*
**Wish to meet a volunteer**				
	No	1.0		
	Yes	13.0	[2.2-72.1]	*0.003*

Multivariate analysis revealed that only the wish of a patient to meet a volunteer was significant (p = 0.003): OR 13.0 [95% CI: 2.2-72.1] (Table 4).

Only six ADs (50.0%) were filed. One person confirmed his ADs three years after writing it, which is the maximum period of legal validity. Life expectancy was significantly shorter for patients who wrote an AD than those who did not or who did not express an opinion. The average lapse of time between the filling in the patient information form and the patient’s death was 1.41 years.

#### Qualitative findings

Content analysis of the six filed ADs by two researchers revealed two main themes: the first comprised wishes regarding medical care, in particular life-sustaining treatment, and was addressed to the haematologist or the family doctor (Participants 2, 3 and 5); the second theme referred to more personal messages the patients wished to express to their relatives regarding their personal philosophy about end of life (Participants 1, 2, 3 and 4). Six key terms emerged from this analysis: "life-sustaining treatment", "my children," "mental faculties", "survival", "suffering" and "pain".

### Them 1: patients’ wishes about medical care decisions

Two patients began their directives by asserting that they were of sound mind and emphasizing how important their state of consciousness was for them:

*In full possession of my mental and physical faculties, such are my directives… I intend to be as conscious as possible at the time of my death…* (Participant 2)

*I, the undersigned Mr B…, being of sound mind if not of sound body, hereby declare…* (Participant 5)

The patients anticipated the possibility that they might lose their faculty of expression:

*A worsening of my state of health*… (Participant 1)

*A deterioration of my faculties…* (Participant 2)

*If the progression of my state suddenly reduces my free will, whatever the cause, I request*… (Participant 5)

All the patients expressed their refusal of life-sustaining care, but only some of them explained what they meant by that term:

*I wish to be spared from all aggressive therapy, as well as all artificial means of survival that would leave me deprived of my mental faculties or suffering from intolerable pain.* (Participant 3)

*I do not want any life-sustaining care…* (Participant 1)

*I refuse all life-sustaining care…* (Participant 4)

*No life-sustaining care, I do not want to be a guinea pig*… (Participant 6)

Some ADs were unequivocal, others were more difficult to interpret. Sometimes a discrepancy appeared between the refusal of life-sustaining treatments and the fact that the patient still agreed to receive some of them:

*I request that my life is not prolonged… I have survived thanks to transfusions for four years… Let the medical profession sort this out themselves. I hereby free them of all civil and penal responsibility*… (Participant 5)

The patients indicated what they agreed to and refused in terms of medical techniques. They mentioned palliative care and pain relief:

*Yes for administration of painkillers, so that the pain will not prevent me from serenely expressing myself…* (Participant 2)

*I wish to be spared from intolerable suffering…* (Participant 3)

*I fear all pain…* (Participant 5)

Two concepts of medicine emerged: palliative treatment providing support for the patient on one hand and technical intervention on the other. This led patients to refuse some treatments perceived as futile or associated with a fear of medicine:

*Yes to palliative care. No to all aggressive care. No to any surgery that would leave the final prognosis unchanged…* (Participant 2)

*Should the possibility arise, I absolutely refuse all resuscitation* (Participant 4)

Sometimes, the patient refused treatments which could lead to disability due to secondary effects:

*No to all drugs likely to induce the loss of my remaining faculties (sight, mobility, bodily functions)…* (Participant 2)

The more explicit AD made references to the patient’s medical history, to his quality of life, perceived as less than satisfying, to the disorders or symptoms he suffered from, to his fears and anxiety:

*My main anxiety is choking and suffocation. My own father died in my home by suffocation… unable to express himself… unconscious… The experience I had that day was too acute for time to erase…* (Participant 5)

*The serious structural weakness, which is worsening as my disease progresses, is taking me from my armchair to my bed to drowse or to sleep…* (Participant 5)

ADs were also a means for the patient to bring up the question of the place of death, or to allude to complex family relationships:

*Following the patient information form I just completed, I inform you that in case of hospitalization and worsening of my state, I do not wish to die in L…*

*I express a very doubtful opinion regarding the information my daughter will be supplied with*… (Participant 1)

### Theme 2: personal messages to relatives or a personal philosophy about end of life

ADs were also used to communicate their thoughts on the meaning of life, personal quest for serenity, and to show how much they trusted their loved ones:

*I have had my time… Life is a marvel but one must know how to end even the best of things. I will have lived enough…* (Participant 5)

*My sincere desire is to end the path of my life with the best support, so that it will reach its conclusion in all serenity…* (Participant 4)

*In case of deterioration of my faculties, I trust my wife and any of my six children to make a decision according to the spirit of the present document…* (Participant 2)

Two patients referred to the use of this document and the implications it could have in making future medical decisions:

*I thank you in advance for the attention you will give to me…* (Participant 4)

*I thank you for agreeing to take these guidelines into consideration…* (Participant 1)

## Discussion

The main findings of this mixed-method approach to better understand which factors influence patients with an HM in using end-of-life tools are that: i. patients with partners are more likely to appoint an HCP and, ii. patients wishing to meet a religious representative or a volunteer are more likely to write an AD. This significant difference in ADs in terms of religious belief has never been described before except for euthanasia requests [8]. Furthermore, this is the first paper to provide a qualitative analysis of the AD content in [19–22] a population of patients with HM. Two main themes emerge: wishes relative to medical treatment on one hand, and personal messages to loved ones on the other.

The patients who designated an HCP in our study were older than those from previously published works presenting with other pathologies [23–25]. The proportion of patients who were married or with a partner in our study was similar to that of patients admitted for surgical procedures (71.0% vs.76.0%, respectively) [26]. However, this figure is much higher than that found in the general French population (47.0%) [27].

None of the factors of gender, age, pathology or expression of a wish to meet a volunteer or a religious representative were found to be a significant influence on whether a patient chooses to designate an HCP. This is in contrast to the findings of Halpern *et al.*[24] who showed that age is a significant factor and that there is a relationship between the designation of an HCP and religion and spirituality. However, it is difficult to compare the studies directly. Demographical data are rarely if ever published at a national level. Furthermore, the concept of ADs in the US [2] is different as the term is used indiscriminately for both ADs and HCPs.

Overall, patient/carer relationships have been changing recently in France towards a model that encourages more patient autonomy. Talking about a subject as sensitive and difficult as end of life is possibly easier with an HCP.

In our study, we found a higher rate of HCP designation (64.5%) than the national rate in France (5.0%) [5] though a similar rate to that found in the US [24]. The main factors for designating an HCP are related to culture and the country, the pathology and also how advanced the society is in dealing with this kind of procedure: percentages vary enormously from nearly 30% in Europe to 0.0% in Japan [28].

Among the reasons potentially explaining HCP designation is the diagnosis of a severe disease, as in our setting, where patients have more of an acute need. Furthermore, the high rate we observed in our population could also be a reflection of our active information programme including patient information documents and workgroups comprising doctors, caregivers, and a jurist specialized in medical law which might have facilitated exchanges on the subject. However, in spite of this multi-step programme introduced in our service as from 2008, there was no increase in the number of patients designating HCPs or writing ADs over the study period.

The most frequently designated HCP were members of the patient’s family (93.0%), which matches other reports found in the literature [16, 19, 26, 28, 29]. French law stipulates that a patient can only designate a single HCP; however, seven of our patients designated several HCPs. This might indicate a difficulty in choosing between children and spouse [19].

The people who wrote ADs tended to be older – 75.5 years as opposed to 65.4 – similarly to other studies finding that older patients are more likely to write ADs [19, 20, 24].

People who wrote an AD were also more likely to express a wish to meet a religious representative and a volunteer simultaneously regardless of practice or belief, or religion (although Catholicism was the only religion mentioned by the patients who expressed a wish to meet a religious representative (56.3%)). The need for spirituality and religious support is described in the literature as playing an important role in palliative care [10, 30–33].

The fact that the ADs were written after diagnosis could suggest that the wishes expressed are more the consequence of the patient’s personal experience rather than a reaction when faced with imminent death.

The desire to meet a volunteer could attest to a need to open up to others. When someone is diagnosed with a severe disease they are forced to face up to the reality of their own mortality and this leads to introspection or contemplation about their relationships with other people and the world in general. This questioning can result in a need for spirituality.

The low number of ADs written by our patients with an HM (6.0%) was similar to that found in a French study carried out in various medical units (6.0%) [15], but is higher than the results of a national French observatory study (2.5%) [33]. These results are close to those observed in Europe (from 2.0% to 18%) and reflect the limited use of ADs despite encouragement through specific legislation [19, 34]. The low figures can be explained by the fact that patients are either not aware of this concept (90.0%) or have a negative perception of it [15, 31, 32]. Conversely, more patients write ADs in the US (from 1.5% to 71.0%) depending on the population studied, and this highlights major cultural differences between Europe and the US [30, 35–39].

Among the registered ADs, two were not archived and the haematologist did not ask the patients to transmit them based on the argument of the anxiety it could trigger. ADs are often assimilated to death, making it difficult for the carers to approach the subject. On the data updated on July 5, 2013, most of the patients who wrote an AD had died (n = 4/6) although the mortality for the whole studied cohort was 27.4%.

All ADs were written on blank paper and not on a pre-printed form and comprised one to two pages. When a patient went into more detail about what they accepted or refused in terms of medical care, they referred to their own experience and gave limits for a specific treatment or determined a degree of handicap that was not acceptable to them [40]. Past family experience of the disease was also referred to with a very accurate description of the symptoms the patient witnessed personally and expressing a desire not to suffer the same fate.

Furthermore, no ADs expressed a wish to have everything done to sustain life regardless of prognosis or cost. Few patients used ADs as a way to ponder on the meaning of life, the end, or to declare his/her spiritual quest.

The people who wrote ADs were able to pursue a personal reflection about their disease, to anticipate their incapacity to express their will and to envisage their own death. They simultaneously designated an HCP (75.0%) suggesting a need to exchange orally as well as in writing. This finding matches other reports in the literature [39, 41].

The finding that so few ADs are written in this patient population would seem to suggest that this tool only responds to the needs and expectations of a minority of patients. This raises questions about the whole of concept ADs and how best to encourage patients to communicate their wishes concerning treatment and how they would like to be supported.

At a time of particular fragility, HCPs and ADs make tangible a confrontation with the idea of our own end. This is particularly important in the society in which we live as the rituals that previously helped us tame our fear of death gradually fade away. This psychological aspect should not be neglected. The Terror Management theory argues that human understanding of mortality creates an existential anxiety that must be kept under constant control. Defences are erected to keep thoughts about death as far removed from the consciousness as possible [42, 43]. Not everybody can face their own death and write about it.

Moreover, the implementation of HCPs and ADs could undermine a type of carer/patient communication which is both oral and based on trust. Informal oral ADs do not exist in the US but represent 11.0% of German ADs [28] while they have not been analysed in France. Trust in relatives and in the medical team is sufficient for some patients who do not see the need to formalize words discussed in private. One publication shows only one patient out of three goes the whole way in the process of writing [44]. It would thus appear essential to respect the will and rhythm of the patient to communicate in his/her own way on such a sensitive subject as the end of life. ADs and HCP should not replace a discussion with the patient who might prefer to express his/her wishes in this way.

### Limitations

Apart from concerns of potential selection bias and confounding factors inherent to any retrospective study, some other limitations deserve to be mentioned. First, it took place in a single centre and thus cannot be generalized to all patients presenting with an HM. Furthermore, similar to other mixed-method studies the qualitative and quantitative approaches took place in a sequential order thus limiting the integration of both data forms under a unified process of data analysis. However, the mixed-method approach is highly relevant when dealing with current public health issues, It allows researchers to combine the strengths of qualitative and quantitative methodologies and can reveal which variables are related, the predictive nature of one variable over another and the characteristics of this predictive relation. Another limitation of the study is the low number of ADs available. Moreover, we did not explore what motivates the patient to choose an HCP and whether HCPs provide a true opportunity for dialogue [45] or rather constitute a mere administrative formality. Gathering the opinions of carers to assess their perception of HCPs and ADs could also have been of interest. These points could be the focus of complementary studies.

## Conclusions

The findings of our study highlight that, despite an advance care planning programme to assist patients and the training of caregivers, few people choose to write ADs. Accompanying a patient through the process of designating an HCP and writing ADs requires time and allocation of support personnel. Both tools are a potential opportunity for starting up a much needed dialogue with a patient facing a life-threatening disease. The fact that only a few patients presenting with severe HM write ADs, would indicate that the need remains to be better defined by French patients. Furthermore, as some patients could find the formal approach of writing difficult, informal oral communication about wishes with carers and relatives should also be encouraged as it represents an opportunity to develop a good doctor/patient relationship.

## Electronic supplementary material

Additional file 1: Table S1: Multi-step procedure initiated in our clinical haematology department to implement the 2005 Patient’s Rights and End-of-Life Care Act. (DOC 178 KB)

## Abbreviations

HCP: Healthcare proxy
AD: Advance directives
HM: Haematological malignancies.
