# Supplementary material for: Factors associated with the designation of a health care proxy and writing advance directives for patients suffering from haematological malignancies
Source: BMC Palliat Care. 2014 Dec 11;13:57. doi: 10.1186/1472-684X-13-57 (PMC4391307; doi:10.1186/1472-684X-13-57)
Supplement: Supplementary file 1 — Additional file 1: Table S1: Multi-step procedure initiated in our clinical haematology department to implement the 2005 Patient’s Rights and End-of-Life Care Act. (DOC 178 KB) [file 12904_2014_249_MOESM1_ESM.doc]

**Additional file 1: Table S1 Multi-step procedure initiated in our clinical haematology department to** **implement the 2005 Patient’s Rights and End-of-Life Care Act**


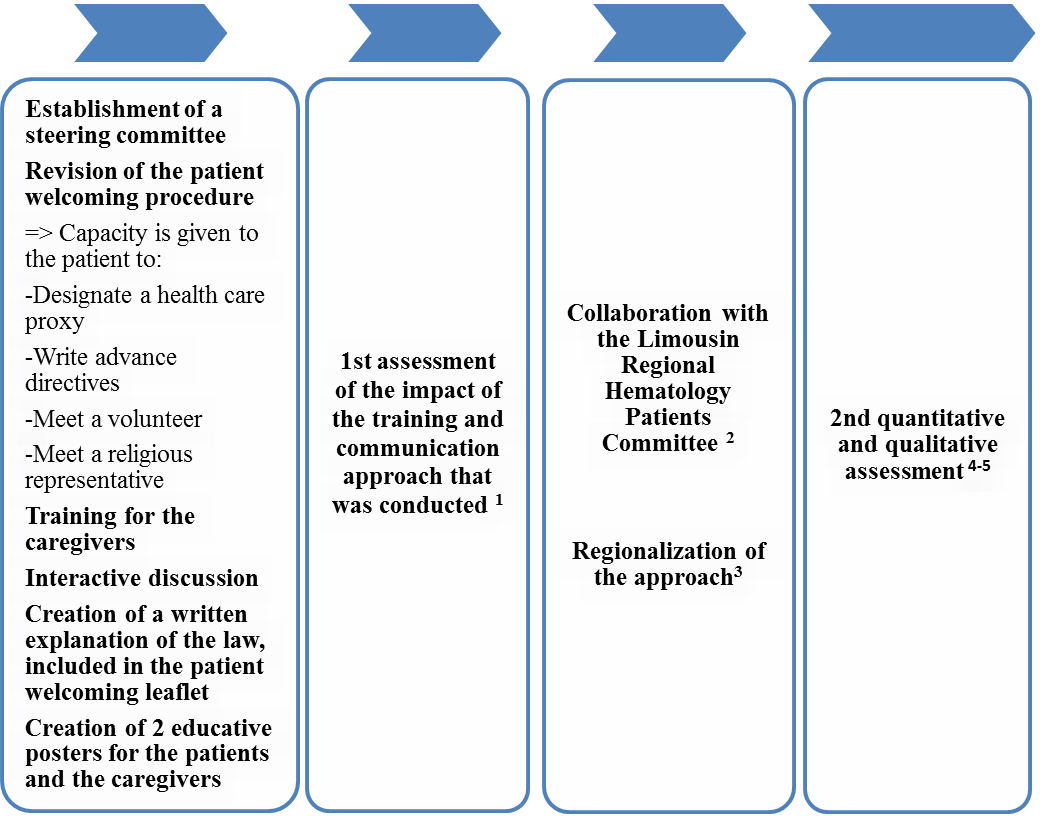


2008

2010

2011

2012/2013

1) *Regard des soignants d’un service d’hématologie clinique sur la loi relative aux droits des malades et à la fin de vie : analyse qualitative et quantitative.* S. Trarieux-Signol, MP. Gourin, J. Guillout, D. Vigier, A. Penot, S. Moreau, D. Bordessoule, communication affichée, 17ème Congrès national d’accompagnement et de soins palliatifs, 2010, Marseille, France.

2) *Regard d’un comité de patients sur la loi dite « Léonetti »,* S. Trarieux-Signol S, L. Castanier, communication orale, 18ème Congrès national d’accompagnement et de soins palliatifs, 2011, Lyon, France.

3) *Le réseau de soins : un maillon essentiel pour l’appropriation des valeurs de la loi « Léonetti »,* S. Trarieux-Signol, M. Touati, M. Denoyer, D. Devesa, J. Venot, P. Lo Re, MP. Gourin, D. Bordessoule, communication affichée, 2ème Congrès national des réseaux de cancérologie, 2011, Rouen, France.

4) *Les directives anticipées facilitent-elles les échanges sur la fin de vie et la mort ?* S. Trarieux-Signol, D. Leboul, S. Moreau, F. Bompart, D. Bordessoule, communication affichée, 19ème Congrès national d’accompagnement et de soins palliatifs, 2013, Lille, France.

5) *Quels rapports les soignants et les patients entretiennent-ils avec la personne de confiance ?* S. Trarieux-Signol, D. Leboul, S. Moreau, F. Bompart, D. Bordessoule, communication affichée, 19ème Congrès national d’accompagnement et de soins palliatifs, 2013, Lille, France.
